# In silico identification of novel ligands targeting stress-related human FKBP5 protein in mental disorders

**DOI:** 10.1371/journal.pone.0320017

**Published:** 2025-03-17

**Authors:** Ovinuchi Ejiohuo, Donald Bajia, Joanna Pawlak, Aleksandra Szczepankiewicz

**Affiliations:** 1 Department of Psychiatric Genetics, Poznan University of Medical Sciences, Poznan, Poland; 2 Molecular and Cell Biology Unit, Poznan University of Medical Sciences, Poznan, Poland; 3 Doctoral School, Poznan University of Medical Sciences, Poznan, Poland; 4 Department of Pediatric Oncology, Hematology, and Transplantology, Poznan University of Medical Sciences, Poznan, Poland; Government College of Engineering, Keonjhar, INDIA

## Abstract

FK506-binding protein 51 (FKBP51 or FKBP5) serves as a crucial stress modulator implicated in mental disorders, presenting a potential target for intervention. Inhibitors like SAFit2, rapamycin, and tacrolimus exhibit promising interactions with this protein. Despite these advances, challenges persist in diversifying FKBP5 ligands, prompting further exploration of interaction partners. Hence, this study aims to identify other potential ligands. Employing molecular docking, we generated complexes with various ligands (rapamycin, tacrolimus, SAFit2-Selective antagonist of FKBP51 by induced fit, ascomycin, pimecrolimus, rosavin, salidroside, curcumin, apigenin, uvaricin, ruscogenin, neoruscogenin, pumicalagin, castalagin, and grandinin). We identified the top 3 best ligands, of which ruscogenin and neoruscogenin had notable abilities to cross the blood-brain barrier and have high gastrointestinal absorption, like curcumin. Toxicity predictions show ruscogenin and neoruscogenin to be the least toxic based on oral toxicity classification (Class VI). Tyrosine (Tyr113) formed consistent interactions with all ligands in the complex, reinforcing their potential and involvement in stress modulation. Molecular dynamic (MD) simulation validated strong interactions between our three key ligands and FKBP5 protein and provided an understanding of the stability of the complex. The binding free energy (ΔG) of the best ligands (based on pharmacological properties) from MD simulation analysis is -31.78 kcal/mol for neoruscogenin, -30.41 kcal/mol for ruscogenin, and -27.6 kcal/mol for curcumin. These molecules, therefore, can serve as therapeutic molecules or biomarkers for research in stress-impacted mental disorders. While offering therapeutic implications for mental disorders by attenuating stress impact, it is crucial to emphasize that these ligands’ transition to clinical applications necessitates extensive experimental research, including clinical trials, to unravel the intricate molecular and neural pathways involved in these interactions.

## Introduction

Stress is recognised as a significant factor contributing to the onset and exacerbation of psychiatric disorders, including bipolar disorder [1]. The intricate interplay between genetic predisposition and environmental stressors can impact the expression of genes associated with regulating stress response [2]. In the context of bipolar disorder, dysregulation of stress-related pathways, including the hypothalamic-pituitary-adrenal (HPA) axis, may contribute to the neurobiological and molecular alterations associated with the disorder [3–5]. The dysregulation of the HPA axis has been observed in individuals with bipolar disorder [6]. Genetic variations and altered expression of stress response-related genes, including *FKBP5*, may influence the sensitivity of the HPA axis to stress hormones such as cortisol, thereby impacting neurons and neural circuits implicated in bipolar disorder [7].

The *FKBP5* gene encodes FK506 binding protein 51 (FKBP51 or FKBP5), a co-chaperone protein involved in the glucocorticoid receptor complex and pivotal in stress responses [3]. This makes FKBP5 a potential target for stress-related mental disorders. The interaction of FKBP5 with recently developed selective ligands such as SAFit2 ([8]), rapamycin, and tacrolimus (FK506), which bind to similar FKBP proteins [8–11], demonstrates promising results in *in vitro* and rodent models [3].

Despite advancements, relying solely on these compounds underscores a limitation in the available chemical starting points for developing FKBP5 ligands and discrepancies in FKBP5 interaction patterns, highlighting the need to validate these interactions further [12]. Rigorous studies are needed to distinguish functionally relevant interactions from experimentally detected interactions that lack functional significance [12]. *In silico* identification offers a valuable approach for screening numerous chemical compounds to identify potential modulators of stress-related human FKBP5 protein.

**Fig 1:**
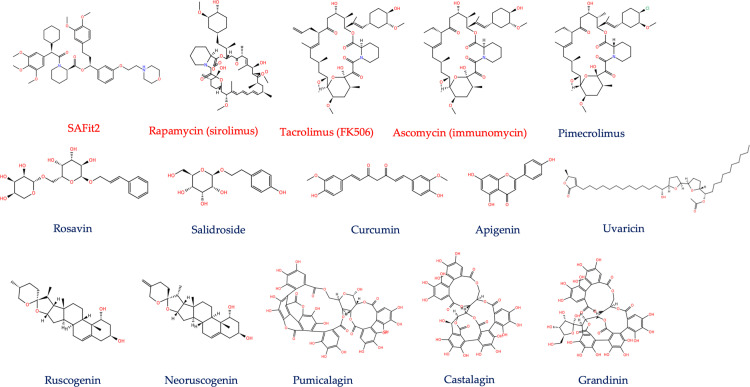
Selected ligands for interactionwith FKBP5 protein [ 13].

This study aims to identify potential ligands that can effectively interact with FKBP5 protein, modulate its activity, and serve as either molecular markers for research studies or offer therapeutic advantages to individuals with stress-related mental disorders in the long run. By employing molecular docking techniques, we assessed the interaction between the ligands depicted in Fig 1 and the human FKBP5 protein, providing an *in silico* rationale for these interactions. Reference ligands, SAFit2, rapamycin, and tacrolimus (FK506), were included in our study for comparative analysis as known inhibitors of the FKBP5 protein. The binding affinity results and interaction visualisations derived from these interactions were utilised to discern the ligands demonstrating efficient interaction with the FKBP5 protein. Molecular dynamic simulation was performed to fully comprehend the stability of the complexes.

## Materials and methods

### Ligand preparation

The Simplified Molecular Input Line Entry System (SMILE) of fifteen molecules: Rapamycin (sirolimus), tacrolimus (FK506), SAFit2 (Selective antagonist of FKBP51 by induced fit), ascomycin (immunomycin), pimecrolimus, rosavin, salidroside, curcumin, apigenin, uvaricin, ruscogenin, neoruscogenin, pumicalagin, castalagin, and grandinin was retrieved using PubChem (https://pubchem.ncbi.nlm.nih.gov/) [14]. Rapamycin, tacrolimus, and SAFit2 are considered reference ligands for this study as they are validated ligands that can inhibit FKBP5 protein [8,15,16] and aim to serve as references for comparison with other chosen ligands. The other ligands were chosen due to their function, structure, or source of origin similarity with the reference ligands. Also, investigating ligands with immune activity is crucial for mental disorders due to the growing evidence linking immune dysfunction to the pathophysiology of psychiatric conditions [17–21]. Because psychosocial stress can cause changes in the nervous and hormonal systems, weakening the immune system and increasing the risk of diseases [22,23], molecules with immunoactivity might also be useful for psychiatric conditions [24], with some, like curcumin, already implicated in psychiatric disorders [25–27]. Including only these 12 ligands ensures a manageable yet representative subset of compounds for docking and analysis. This targeted selection improves the chances of identifying high-affinity ligands while reducing redundancy. Limiting the number of ligands also allows for a focus on detailed computational analysis, further ensuring high-quality results. Table 1 below provides a summary basis for the selection of the ligands. Molecular docking and simulation processes can be computationally intensive. Selecting only these 12 ligands balances computational resource requirements but ensures diverse potential inhibitors coverage. Corina Classic 3D server [28–30] was then used to generate the 3D structures in PDB file format for the docking process.

**Table 1:** Selection of ligands for the study.

	Ligand		Justification for selection	References
Reference ligand				
1	SAFit2	Synthetic drug	Validated ligand that selectively inhibit FKBP5 Involved in depression, anxiety, addiction	[31–34]
2	Rapamycin	Microbial origin	Direct or indirect validated ligand that inhibits FKBP5 Immunosuppressive and biological activity	
3	Tacrolimus (FK506)			[35–38]
ligands of microbial origin				
4	Ascomycin		Immunosuppressive and biological activity	[39–42]
5	Pimecrolimus			
Natural plant products				
6	Rosavin		Anti-depressive like effect, effect on depression, environmental stress response, antioxidant, neuroprotective	[43–47]
7	Salidroside		Neuroprotective, anti-inflammatory	[48–52]
8	Curcumin		Antioxidant, anti-inflammatory, neuroprotective	[25–27]
9	Apigenin		Sedative, anti-inflammatory, neuroprotective	[53–57]
10	Uvaricin		Antitumor activity with neurotoxic effect (less studied therapeutic use)	[58,59]
11	Ruscogenin		Steroidal saponins with anti-inflammatory potential and other biological activities (less studied therapeutic use)	[60–64]
12	Neoruscogenin		Steroidal saponins with anti-inflammatory potential (less studied therapeutic use)	[65–67]
13	Pumicalagin		Ellagitannins involved in breast cancer associated with bipolar disorder	
14	Castalagin			[68]
15	Grandinin			

### Protein preparation and quality check

The 3D protein structure of human FKBP5 protein was obtained from RCSB Protein Data Bank (PDB ID: 5OMP) in PDB format [69]. The binding site of the protein and the binding site residues were identified using PrankWeb [70–72]. The protein structure quality was validated using Verify 3D [73–75] and ERRAT [74] on the SAVESv6.0 (structural validation server).

### Molecular docking

Molecular docking was performed using AutoDock version 4.2.6 [76]. Water molecules and heteroatoms were removed from the protein, and polar hydrogens and Kollman charges were added. Hydrogens and charges were also added to the ligand molecule. Torsion bonds were checked and limited to a maximum of 32. The Grid box axis parameters were adjusted to the protein’s active site, defined by coordinates generated from PrankWeb. Docking parameters, including the genetic algorithm, were standardized to 50 runs and a population size of 300 for all ligand-protein complexes, ensuring uniformity across simulations.

### Post-docking analysis of ligand-protein complex

Post-docking analysis of our ligand-protein complex was carried out using various servers. Molegro Molecular Viewer (http://molexus.io/molegro-molecular-viewer/) [77] was used to visualize the 3D ligand-protein complex and generate the interaction surfaces. Protein Plus (https://proteins.plus/) [78], pose view was used to generate 2D interaction diagrams of the binding poses of the complex as well as 3D positioning of the ligand within the binding site of the protein. A 2D interaction diagram from LigPlot Plus [79] further validated the interaction.

### Ligand properties screening

SwissADME [13] was used to access the ligands’ pharmacological and ADME (absorption, distribution, metabolism, and excretion) properties. The predicted toxicity of the ligands was determined using ProTox 3.0 and STopTox [80,81] according to the methods described in our previous study [82].

### Molecular dynamic simulation

Molecular dynamic (MD) simulation of the ligand-protein complex was conducted using SiBioLead (https://sibiolead.com). The binding free energy of the ligand was calculated using the Molecular Mechanics Poisson-Boltzmann Surface Area (MMPBSA). The complex was placed in a triclinic box with Simple Point Charge (SPC) water containing 0.15 M NaCl and carried out using the Optimized Potentials for Liquid Simulations-all atoms (OPLS/AA) forcefield. The system was energy-minimized using the Steepest Descent integrator for 5000 steps. Equilibration was carried out at a temperature of 300K and pressure of 1 bar for 100 ps. The simulation parameter used the Leap Frog integrators with a simulation time of 100 ns with 5000 frames saved. The simulation result analysis was then carried out. Results from the simulation (root mean square deviation, root mean square distribution, root mean square fluctuation, radius of gyration, solvent-accessible surface area, hydrogen bonds) were visualized using QtGrace Version 0.2.7 software according to the methods described in our previous study [82].

## Results and discussion

### Protein structure validation

**Fig 2:**
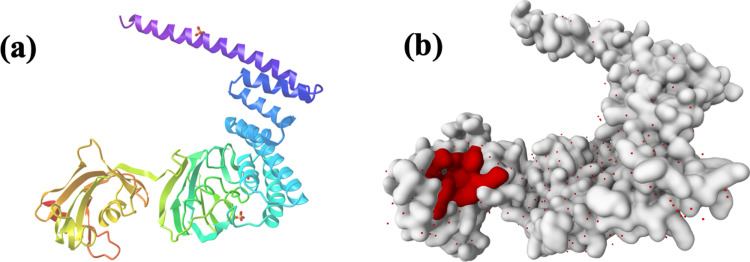
Human FKBP5 protein [ 69]. (a) Ribbon diagram of the 3D structure showing the 3 domains. Yellow-FK1 domain; greenish blue-FK2 domain; bluish purple-TPR domain [83]. (b) Binding pocket 1 showing the area with surface residues [70–72].

PrankWeb result returned two pockets for FKBP5 protein (Fig 2a). Pocket 1 (active binding site) in Fig 2b had a higher score of 2.79 and a probability of 0.087 than Pocket 2 (allosteric site), with a score of 1.84 and a probability of 0.034. Eleven residues were predicted to make up the active binding pocket (pocket 1) of the protein: TYR57, PHE67, ASP68, PHE77, GLN85, VAL86, ILE87, TRP90, TYR113, ILE122, PHE130. This pocket has 22 surface atoms and x, y, and z coordinates of 10.1421, -16.0483, and -29.7898, respectively.

The structural component of the FKBP5 protein is the N-terminal FK1 domain, which corresponds to pocket 1 in the PrankWeb findings and binds immunosuppressive ligands such as tacrolimus and rapamycin. The FK1 domain of FKBP proteins is associated with its regulatory function for glucocorticoid receptor (GR) [84]. A second domain that is not bound to these ligands is the FK2 domain (pocket 2). A third domain called tetratricopeptide repeat (TPR) also exists [83].

**Fig 3:**
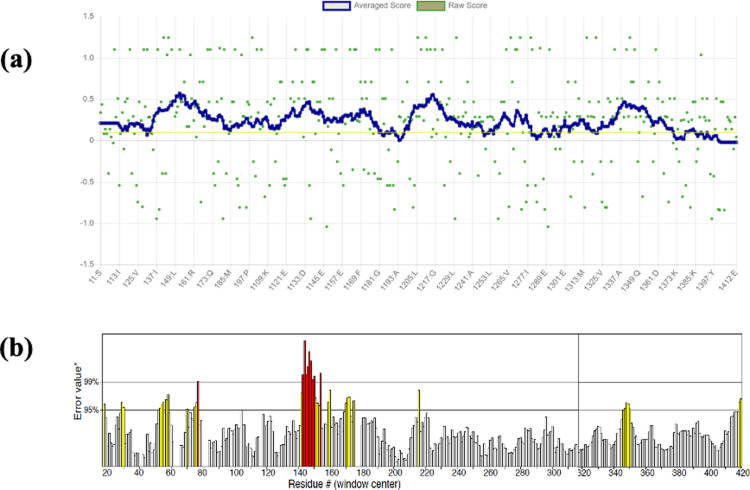
FKBP5 protein quality check result (a) Result from Verify3D (b) ERRAT result. * On the error axis, two lines indicate the confidence with which regions that exceed that error value can be rejected. **Expressed as the percentage of the protein for which the calculated error value falls below the 95% rejection limit. Suitable high-resolution structures generally produce values around 95% or higher. For lower resolutions (2.5 to 3A), the average overall quality factor is around 91%.

The protein structure passed the Verify3D check, with 84.95% of the residues having an averaged 3D-1D score greater than or equal to 0.1, as shown in Fig 3a below. Fig 3b shows the result from ERRAT check with an overall quality factor** of 89.844. Although the highest quality structures have values between 95% and 99%, the average quality factor is around 91%, and our FKBP5 protein structure with 89.844% is around this average, making it a suitable quality structure.

### Docking analysis of the ligand-protein complex

The binding energies from the AutoDock predictions were between -8.21 and -3.59 kcal/mol, which was lower than the binding energies of the reference ligands SAFit2 (-5.78 kcal/mol) and Tacrolimus (-7.11 kcal/mol) (Table 2).

**Table 2:** Ligand-protein docking result with FKBP5 protein.

Ligand	RMSD (Å)	Binding Affinity (kcal/mol)	Inhibition Constant (Ki)	No. of Conformations	Entropy of Conformation
SAFit2	29.74	-5.78	57.90 uM	1	0.98
Rapamycin	26.35	-8.45	643.89 nM	7	0.65
Tacrolimus	27.75	-7.11	6.17 uM	23	0.39
**Ascomycin**	**27.83**	**-7.47**	**3.35 uM**	**10**	**0.57**
**Pimecrolimus**	**27.96**	**-8.21**	**954.29 nM**	**17**	**0.52**
Rosavin	31.36	-4.83	287.87 uM	3	0.82
Salidroside	32.43	-5.20	153.11 uM	5	0.68
**Curcumin**	**32.16**	**-6.74**	**11.53 uM**	**22**	**0.37**
Apigenin	31.63	-5.51	91.87 uM	12	0.34
Uvaricin	31.80	-3.59	2.32 mM	1	0.99
**Ruscogenin**	**28.85**	**-7.68**	**2.35 uM**	**23**	**0.18**
**Neoruscogenin**	**28.72**	**-7.64**	**2.50 uM**	**10**	**0.32**
**Pumicalagin**	**26.71**	**-6.23**	**27.19 uM**	**9**	**0.59**
Castalagin	28.27	-4.33	673.07 uM	5	0.60
Grandinin	29.65	-3.81	1.62 mM	15	0.58

The docking result and analysis of the ligand-FKBP5 protein are presented in Table 2 below.

Binding free energy (ΔG), or in the case of AutoDock results, binding affinity is a measure of the strength of the ligand-protein interaction, and the more negative binding energy or binding affinity, the more favorable the binding interaction [85]. There is, however, no universal threshold indicating superior interaction or inhibition, but the more negative these numbers are, the stronger the interaction, the more favorable the conformational changes, and the more stable the ligand-protein complex. In selecting our best-interacting ligand, we established our criteria based on the magnitude of the binding affinity, the inhibitory constant Ki, which is the maximum inhibitory constant at which 50% of the protein is inhibited, and the number of conformational changes. It is important to note that the more negative the binding affinity, the smaller the Ki, suggesting an inverse relationship as observed from Table 2 above. The number of conformations represents various possible spatial arrangements or shapes the ligand assumes when interacting with a protein. A higher number of conformations provides a more comprehensive exploration of the ligands’ binding possibilities to the protein’s active site, aiding in identifying potential binding modes. Ruscogenin and tacrolimus had the highest conformations (23), followed by curcumin with 22.

The best binding pose for our protein-ligand complex is the one which has the top scoring value (kcal/mol), that is, the lowest affinity energy after going through several binding possibilities or conformations. As such, this implies that the highest conformation does not mean the best binding pose. Based on these criteria, the lowest binding affinity was for rapamycin (-8.45 kcal/mol) followed by pimecrolimus (-8.21 kcal/mol), ruscogenin (-7.68 kcal/mol), neoruscogenin (-7.62 kcal/mol), ascomycin (-7.47 kcal/mol), tacrolimus (-7.11 kcal/mol), curcumin (-6.74 kcal/mol), and pumicalagin (-6.23 kcal/mol). All eight ligands had lower binding affinities than the reference ligand SAFit2 with -5.78 kcal/mol. The more stable the ligand-protein interaction, the lower the entropy of the conformation and the more efficient the binding. It is important to note that the docking results serve only as a valuable computational prediction of the ligand-protein interaction. Experimental investigations such as X-ray crystallography or nuclear magnetic resonance (NMR) are needed to further validate the ligands’ inhibitory activity on the protein.

### Pharmacokinetic and toxicity properties of the ligands

Lipinski’s rule of five (RO5), also called Pfizer’s rule of five, was used to analyze the SwissADME results. According to this rule, the hydrogen bond acceptor should not be greater than ten, the hydrogen bond donor should not be greater than five, the molecular weight should not be greater than 500 g/mol, and the octane-water partition coefficient (logP) should not be greater than five [86]. This information for all ten ligands is available in Table 3 below.

**Table 3:** ADME parameters and pharmacokinetic properties of the ligands. LogP_o/w_ is the average of all lipophilicity predictions in S1 Table. It is a common descriptor of lipophilicity [13].

Ligand	Molecular weight (g/mol)	H bond acceptor	H bond donor	LogP_o/w_	LogS (solubility class)	GI absorption	BBB permeant	Bioavailability score
SAFit2	802.99	11	0	6.46	Insoluble	Low	No	0.17
Rapamycin	914.17	13	3	4.57	Moderately soluble	Low	No	0.17
Tacrolimus	804.02	12	3	3.59	Moderately soluble	Low	No	0.17
Ascomycin	792.01	12	3	3.45	Moderately soluble	Low	No	0.17
Pimecrolimus	810.45	11	2	4.45	Moderately soluble	Low	No	0.17
**Curcumin**	**368.38**	**6**	**2**	**3.03**	**Moderately soluble**	**High**	**No**	**0.55**
Apigenin	270.24	5	3	2.11	Moderately soluble	High	No	0.55
**Ruscogenin**	**430.62**	**4**	**2**	**4.23**	**Soluble**	**High**	**Yes**	**0.55**
**Neoruscogenin**	**428.60**	**4**	**2**	**4.01**	**Soluble**	**High**	**Yes**	**0.55**
Pumicalagin	1084.72	30	17	0.01	Moderately soluble	Low	No	0.17

Curcumin, apigenin, ruscogenin, and neoruscogenin obeyed all 5 Lipinski druglikeness rules for oral bioavailability (a full breakdown is available in S1 Table). SAFit2, rapamycin, tacrolimus, ascomycin, and pimecrolimus violated two (molecular weight greater than 500 g/mol and number of hydrogen bond acceptors greater than 10). Pumicalagin also violated these two, including its number of hydrogen bond donors greater than 5. According to the study by Benet et al. 2016, drugs that violate two or more are usually natural products, natural product derivatives, and non-oral drugs [87]. However, our best-interacting ligands are natural plant products. Other rules for accessing bioavailability are presented in supplementary S1 Table. The Lipinski rule is used to assess the oral bioavailability of molecules, and a violation of two or more indicates that the molecule has poor solubility, poor permeability, or both [87,88]. This means that such molecules will have poor bioavailability. From our results, only curcumin, apigenin, ruscogenin, and neoruscogenin had a bioavailability score of 0.55 each, surpassing the other six ligands, which scored 0.17 each. That is, 55% of these compounds will be absorbed for every gram ingested. The same bioavailability score was obtained for camptothecin analogue FL118 [10,11-Methylenedioxy-20(RS)-camptothecin] and irinotecan in the study by Bagia and Derwish, 2023 [89]. This is particularly important as it has been noted that 50% of drugs being developed fail due to poor oral bioavailability [90]. Our analysis also highlights the ease with which each ligand can be synthesized. The synthetic accessibility score (1 being easy and 10 being very difficult) shows apigenin with a score of 2.96 to be the best performer, followed by curcumin (2.97), ruscogenin (7), and neoruscogenin (7). The worst score was for rapamycin, with 10, followed by tacrolimus, with 9.72.

**Fig 4:**
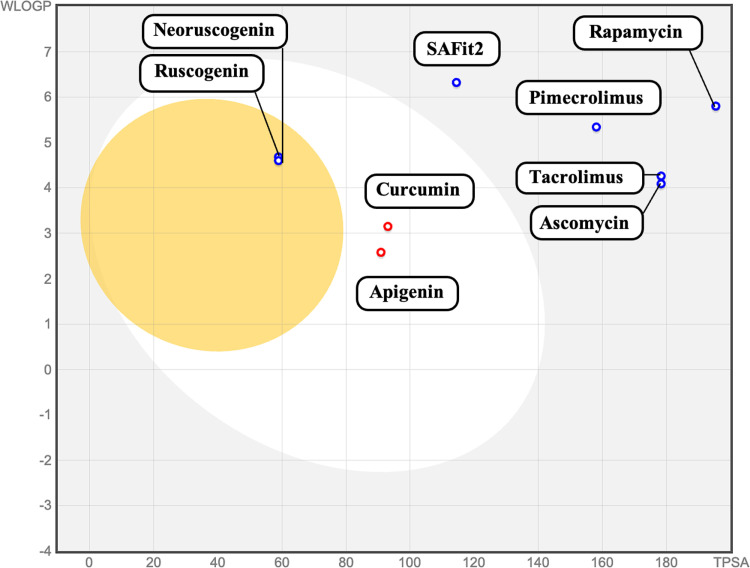
BOILED-Egg representation of the ligands showing their permeation. The yellow circle represents the Blood-Brain Barrier (BBB), the white circle represents Human Intestinal Absorption (HIA), the blue circle PGP +  substrate permeability, and the red circle represent PGP – substrate permeability. WLOGP (William and Crippen Log P). TPSA (Topological Polar Surface Area).

Brain Or IntestinaL EstimateD permeation method (BOILED-Egg) for our selected ligands is presented in Fig 4 below. The model shows the gastrointestinal absorption and brain access of the ligands by calculating the lipophilicity and polarity of the ligands [91].

Ruscogenin and neoruscogenin are predicted to have high gastrointestinal absorption and are the only ligands to permeate the Blood-Brain Barrier, performing better than all other ligands in the group (Fig 4). The ability of both ligands to cross the blood-brain barrier is important for their interaction with the FKBP5 protein in the brain. FKBP5 is present in the hippocampal regions that express the glucocorticoid receptor (GR) [92]. The study by Sharf et al. 2011 on post-mortem mouse brains investigated both RNA and protein levels of FKBP5 in the prefrontal cortex, indicating its presence in the brain [92]. The ability of these ligands to cross BBB means they can directly access the brain and target the FKPB5 protein. Mental illnesses such as bipolar disorder often involve molecular targets within the brain [93]. For instance, voltage-gated calcium channels, specifically the L-type (LTCCs), have been implicated as a molecular target for bipolar disorder [94]. Therefore, ruscogenin and neoruscogenin are more likely to reach these targets, enhancing their therapeutic efficacy. The BBB permeability of these two ligands also ensures that adequate concentrations reach the brain, allowing for sustained and effective interaction with FKBP5 protein. This also means they will have an enhanced ability to reach the brain, reducing the risk of affecting peripheral tissues. In general, both ligands will have improved bioavailability in the brain. However, it is important to note that these ligands will also be able to reach other tissues, necessitating biodistribution studies to ascertain where these ligands predominantly accumulate.

According to our BOILED-Egg model, curcumin and apigenin have similar high gastrointestinal absorption (Fig 4), like ruscogenin and neoruscogenin. Their ability to be absorbed by the human intestine is crucial in their pharmacokinetics and overall bioavailability. This absorption allows these ligands to enter the bloodstream, facilitating systemic distribution to tissues, including the brain [95]. The gut and the brain are connected through the autonomic nervous system, the hypothalamic-pituitary-adrenal axis (HPA), and nerves in the gastrointestinal tract, thus allowing the gut to impact mood, cognition, and mental health [96]. Efficient absorption ensures these ligands are available for distribution to the central nervous system. Molecules with good oral bioavailability are often preferred due to the convenience of oral administration, which can improve patient adherence to treatment regimens [97].

The toxicity prediction result of the ligands in Table 3 is presented in Tables 4 and 5. Table 4 shows the toxicity classification of the ligands. Toxicity classification, based on LD50 values in mg/kg body weight, categorizes substances from Class I (extremely toxic, LD50 ≤  5) to Class VI (non-toxic, LD50 >  5000) according to the Globally Harmonized System (GHS) [80,98,99]. Ruscogenin and neoruscogenin are the least toxic compounds, falling under toxicity Class VI (non-toxic) with LD50 values of 8000 mg/kg and 5000 mg/kg, respectively. Their toxicity profiles are more favorable compared to the reference ligands SAFit2 (Class IV), rapamycin (Class V), and tacrolimus (Class III), indicating a significantly lower potential for adverse effects. This significantly reduces the likelihood of adverse effects compared to the reference ligands, making them promising candidates for further development and clinical use in therapeutic applications targeting FKBP5 protein.

**Table 4:** Oral toxicity classification of the ligands.

Ligands	Toxicity class	LD50 (mg/kg)	Prediction accuracy (%)
SAFit2	4	500	68.07
Rapamycin	5	2500	100
Tacrolimus	3	134	100
Ascomycin	3	134	72.90
Pimecrolimus	5	2500	69.26
Curcumin	4	2000	100
Apigenin	5	2500	70.97
Ruscogenin	6	8000	72.90
Neoruscogenin	6	6000	72.90
Pumicalagin	5	5000	54.26

**Table 5:** Toxicity prediction of the ligands.

		Prediction (probability)									
Classification	Target	SAFit2	Rapamycin	Tacrolimus	Ascomycin	Pimecrolimus	Curcumin	Apigenin	Ruscogenin	Neoruscogenin	Pumicalagin
**Organ toxicity**	Hepatotoxicity	Inactive (0.90)	Inactive (0.87)	Inactive (0.87)	Inactive (0.87)	Inactive (0.91)	Inactive (0.61)	Inactive (0.68)	Inactive (0.85)	Inactive (0.86)	Inactive (0.85)
	Neurotoxicity	Active (0.74)	Active (0.95)	Active (0.95)	Active (0.93)	Active (0.92)	Inactive (0.81)	Inactive (0.86)	Inactive (0.89)	Inactive (0.88)	Inactive (0.90)
**Toxicity end points**	Carcinogenicity	Inactive (0.52)	Active (0.50)	Inactive (0.50)	Inactive (0.54)	Inactive (0.57)	Inactive (0.84)	Inactive (0.62)	Active (0.99)	Inactive (0.60)	Inactive (0.65)
	Immunotoxicity	Active (0.99)	Active (0.99)	Active (0.99)	Active (0.99)	Active (0.99)	Active (0.92)	Inactive (0.99)	Inactive (0.91)	Active (0.99)	Inactive (0.69)
	Mutagenicity	Inactive (0.76)	Inactive (0.74)	Inactive (0.70)	Inactive (0.74)	Inactive (0.69)	Inactive (0.88)	Inactive (0.57)	Inactive (0.75)	Inactive (0.90)	Inactive (0.61)
	Cytotoxicity	Inactive (0.69)	Inactive (0.69)	Inactive (0.64)	Inactive (0.66)	Inactive (0.65)	Inactive (0.88)	Inactive (0.87)	Inactive (0.67)	Inactive (0.75)	Inactive (0.80)
**Tox21-Stress response pathways**	Nuclear factor (erythroid-derived 2)-like 2/antioxidant responsive element (nrf2/ARE)	Inactive (0.96)	Inactive (0.92)	Inactive (0.92)	Inactive (0.92)	Inactive (0.89)	Active (1.0)	Inactive (0.99)	Inactive (0.67)	Inactive (0.66)	Inactive (0.92)
	Heat shock factor response element (HSE)	Inactive (0.96)	Inactive (0.92)	Inactive (0.92)	Inactive (0.92)	Inactive (0.89)	Active (1.0)	Inactive (0.99)	Inactive (0.67)	Inactive (0.66)	Inactive (0.92)
	Mitochondrial Membrane Potential (MMP)	Inactive (0.90)	Active (0.98)	Active (1.0)	Active (0.83)	Inactive (0.73)	Active (1.0)	Active (1.0)	Active (0.57)	Active (0.51)	Inactive (0.71)
	Phosphoprotein (Tumor Su-pressor) p53	Inactive (0.90)	Inactive (0.89)	Inactive (0.93)	Inactive (0.92)	Inactive (0.94)	Active (1.0)	Active (1.0)	Inactive (0.89)	Inactive (0.89)	Inactive (0.57)
	ATPase family AAA do-main-containing protein 5 (ATAD5)	Inactive (0.97)	Inactive (0.98)	Inactive (0.96)	Inactive (0.98)	Inactive (0.98)	Inactive (0.94)	Active (0.96)	Inactive (0.93)	Inactive (0.93)	Inactive (0.97)
**Cytochrome**	CYP1A2	Inactive (0.81)	Inactive (0.98)	Inactive (0.97)	Inactive (0.96)	Inactive (0.96)	Inactive (0.78)	Active (1.0)	Inactive (0.95)	Inactive (0.95)	Inactive (0.89)
	CYP2C19	Inactive (0.69)	Inactive (0.91)	Inactive (0.88)	Inactive (0.91)	Inactive (0.85)	Active (0.94)	Active (0.99)	Inactive (0.94)	Inactive (0.93)	Inactive (0.88)
	CYP2C9	Active (0.51)	Inactive (0.76)	Inactive (0.70)	Inactive (0.78)	Inactive (0.59)	Active (0.89)	Active (0.81)	Inactive (0.83)	Inactive (0.79)	Inactive (0.56)
	CYP2D6	Inactive (0.65)	Inactive (0.83)	Inactive (0.82)	Inactive (0.81)	Inactive (0.76)	Inactive (0.81)	Inactive (0.89)	Inactive (0.87)	Inactive (0.86)	Inactive (0.87)
	CYP3A4	Active (0.66)	Inactive (0.78)	Inactive (0.75)	Inactive (0.78)	Inactive (0.77)	Active (0.63)	Active (0.99)	Inactive (0.81)	Inactive (0.80)	Inactive (0.80)
	CYP2E1	Inactive (1.0)	Inactive (0.99)	Inactive (0.99)	Inactive (0.99)	Inactive (0.98)	Inactive (1.0)	Inactive (0.98)	Inactive (0.99)	Inactive (0.99)	Inactive (0.99)
**Acute inhalation toxicity**		No	No	No	No	No	No	No	No	No	No
**Acute oral toxicity**		Yes	Yes	Yes	Yes	Yes	No	No	Yes	Yes	No
**Acute dermal tox-icity**		No	No	No	No	No	No	Yes	No	No	Yes
**Eye irritation and corrosion**		Yes	Yes	Yes	Yes	Yes	No	Yes	No	No	Yes
**Skin sensitization**		No	No	No	No	No	Yes	Yes	No	No	No
**Skin irritation and corrosion**		No	No	No	No	No	No	No	No	No	No

The toxicity information is important in understanding drug interactions and optimising the ligands’ therapeutic outcomes [82]. From Table 5, Pumicalagin showed no toxicity except positive for acute dermal toxicity and eye irritation and corrosion. Ruscogenin was active for carcinogenicity and Mitochondrial Membrane Potential (MMP) and positive for acute oral toxicity only. Neoruscogenin showed similar patterns as ruscogenin, being active for immunotoxicity and MMP and positive for acute oral toxicity only. Curcumin was active for immunotoxicity, the stress response pathways except ATPase family AAA domain-containing protein 5 (ATAD5), active for three of the six cytochrome enzymes, and positive for only skin sensitization. The toxicity profile of the ligands serves as a crucial safety assessment for advancing them as potential pharmacotherapeutic agents rather than invalidating them [82]. It provides critical information to refine their design, dosage, and delivery mechanisms for safer pharmacological applications. The active toxicity mechanisms, such as mitochondrial membrane potential disruption, immunotoxicity, and cytochrome enzyme activity, indicate specific pathways to address during ligand modification, ensuring enhanced safety and efficacy. Ligands with more toxic profiles may require structural modifications or alternative approaches before further development.

### Analysis of the ligand-protein interactions and conformation

The complexes’ post-docking processing results showed that they had a variety of noncovalent interactions, such as hydrogen (H) bonding and hydrophobic interactions, controlled by interatomic contacts. The number and strength of hydrogen bonds in a ligand-protein interaction can influence the overall binding affinity [100,101]. Hydrogen bonds contribute to the stability of the formed complexes [102]. More hydrogen bonds can enhance the binding affinity by increasing the overall interaction energy [100]. It is, however, also essential to consider the length of the hydrogen bond. Shorter bond lengths significantly contribute to the overall binding energy [103]. A more arranged set of few strong hydrogen bonds may also be more effective than numerous weaker ones. Hydrophobic interactions with the protein pockets also compensate for fewer hydrogen bonds [104]. These interactions may lead to a more stable protein structure through rearrangements that bury hydrophobic surfaces. Ionic interactions and other electrostatic forces also contribute to the overall binding strength [105,106] and may not involve hydrogen bonds directly.

**Fig 5:**
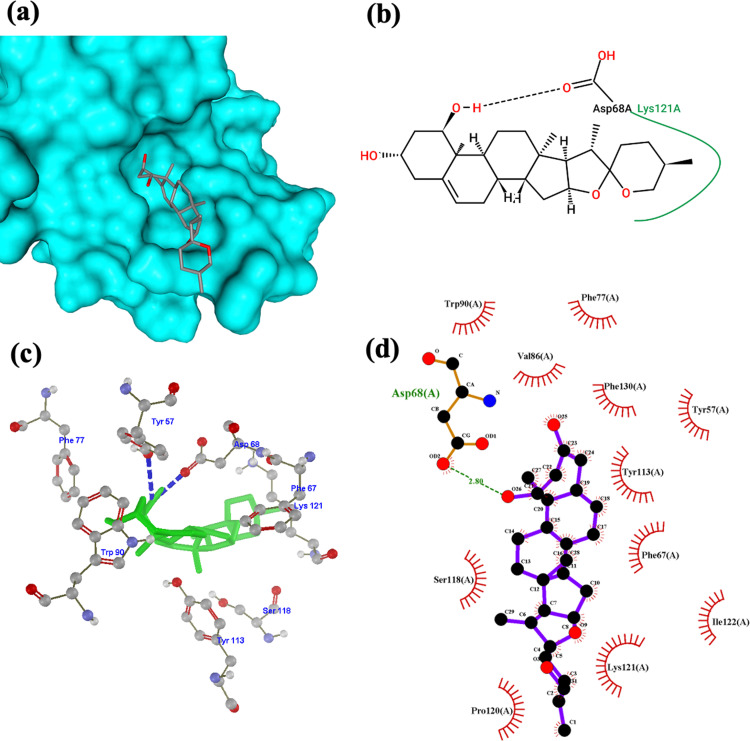
Ruscogenin-FKBP5 complex visualization. (a) 3D structural orientation of the complex from Protein Plus (b) 2D binding pose interaction of the complex from Protein Plus. Black dash lines represent H bonds, green lines represent hydrophobic interaction (c) 3D arrangement of the complex from Molegro Molecular Viewer. Blue dash lines represent H bonds (d) 2D visualization of the interaction from LigPlot. The green text represents the interacting residue, and the green dashed lines represent the H bond.

In Fig 5b–5d, ruscogenin formed hydrogen bonds with Asp68 with a bond length of 2.80 in (d). An additional hydrogen bond is formed with Tyr57 in (c). Fig 5b shows the hydrophobic interaction of the ligand with Lys121. Results from the Protein-Ligand Profiler show two hydrogen bonds: Asp68 with hydrogen-acceptor bond (H-A) distance of 1.85, donor-acceptor (D-A) distance of 2.80 and Lys121 (HA:3.38) and (DA:4.02). The following hydrophobic interactions and respective distances were obtained: Tyr57 (3.37), Phe67 (3.42), Trp90 (3.911), Tyr113 (3.48), Pro120 (3.74), Lys121 (3.50), Ile122 (3.83), and Phe130 (3.60). This result suggests that the predicted H bond with Tyr57 is possible rather than a hydrophobic interaction.

**Fig 6:**
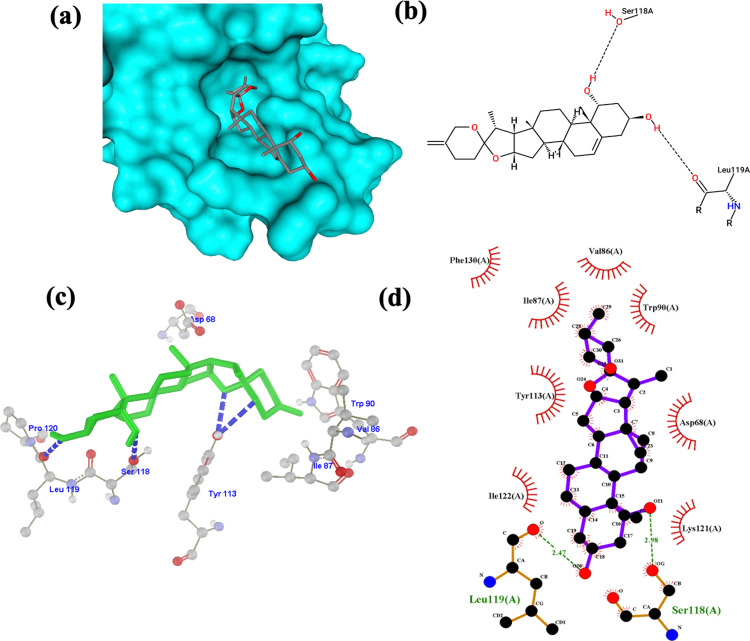
Neoruscogenin-FKBP5 complex visualization. (a) 3D structural orientation of the complex from Protein Plus (b) 2D binding pose interaction of the complex from Protein Plus. Black dash lines represent H bonds, green lines represent hydrophobic interaction (c) 3D arrangement of the complex from Molegro Molecular Viewer. Blue dash lines represent H bonds (d) 2D visualization of the interaction from LigPlot. The green text represents the interacting residue, and the green dashed lines represent the H bond.

In Fig 6b–6d, neoruscogenin formed hydrogen bonds with Ser118 with a bond length of 2.98 in (d) and Leu119 with a bond length of 2.47 in d. An additional hydrogen bond is formed with Tyr113 in (c). Results from the Protein-Ligand Profiler show two hydrogen bonds: Ser118 with hydrogen-acceptor bond (H-A) distance of 2.16, donor-acceptor (D-A) distance of 2.98 and Leu119 (HA:1.88) and (DA:2.47). The following hydrophobic interactions and respective distances were obtained: Val86 (3.45), Ile87 (3.08), Trp90 (3.42), Trp90 (3.03), Lys121 (3.82), Ile122 (3.66), and Phe130 (3.84).

Ruscogenin and neoruscogenin are steroidal saponins first isolated from *Ruscus aculeatus* (butcher’s broom) [107]. Neoruscogenin showed an affinity for retinoid-related orphan receptor α (RORα) (NR1F1) [67], whose biological function is unknown, but the anti-inflammatory potential was suggested [108]. In the study by Bi et al. 2013, ruscogenin treatment significantly decreased leukocyte infiltration and the expression of inflammatory cytokines in rats [61]. Ruscogenin and neoruscogenin have anti-inflammatory properties, and chronic inflammation is linked to stress-related disorders [109]. The study by Maydych 2019, noted that the onset, persistence, and recurrence of depression are significantly influenced by inflammatory activity and affective-cognitive alterations that are triggered by psychological stress [110]. Therefore, if these molecules reduce inflammation, they may positively impact stress-related mental disorders. Literature and research on the therapeutic potential of saponins in mental disorders, in general, are hardly available [111], especially of ruscogenin and neoruscogenin, whose neuroprotective and overall therapeutic potential are yet to be investigated. The study by Chung et al. 2002, however, demonstrated that polygalasaponin had antagonistic activity against dopamine and serotonin in vivo[112], making it a potential antidepressant agent. Both neurotransmitters (dopamine and serotonin) play crucial roles in mood regulation associated with depressive disorders. This might hold promise for ruscogenin and neoruscogenin being used for similar functions.

**Fig 7:**
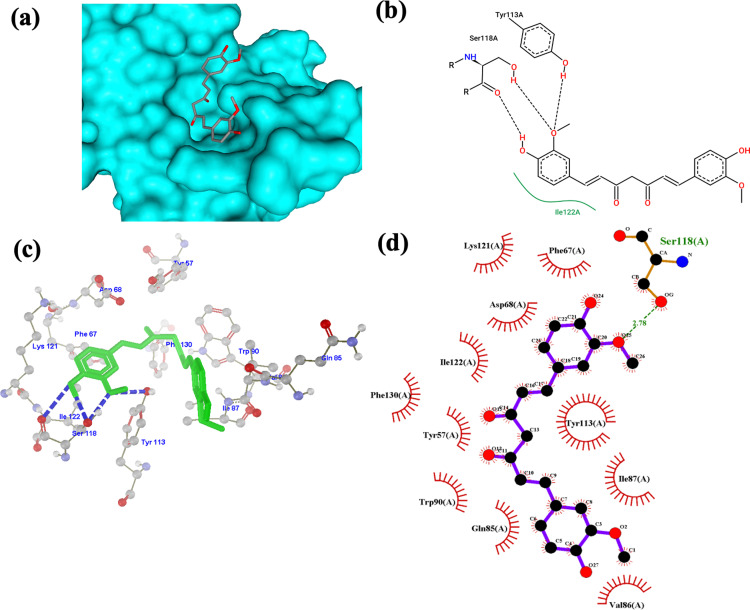
Curcumin-FKBP5 complex visualization. (a) 3D structural orientation of the complex from Protein Plus (b) 2D binding pose interaction of the complex from Protein Plus. Black dash lines represent H bonds, green lines represent hydrophobic interaction (c) 3D arrangement of the complex from Molegro Molecular Viewer. Blue dash lines represent H bonds (d) 2D visualization of the interaction from LigPlot. The green text represents the interacting residue, and the green dash lines represent the H bond.

In Fig 7b–7d, curcumin formed hydrogen bonds with Ser118 with a bond length of 2.78 in d and Tyr113 in (b) and (c). An additional hydrogen bond is formed with Ile122 in (c). Fig 7b shows the hydrophobic interaction of the ligand with Ile122. Results from the Protein-Ligand Profiler show one hydrogen bond with two distances: Ser118 with hydrogen-acceptor bond (H-A) distance of 2.18 and 1.81, donor-acceptor (D-A) distance of 3.14 and 2.78. The following hydrophobic interactions and respective distances were obtained: Phe67 (3.19), Phe67 (3.18), Ile87 (3.90), Ile87 (3.77), Trp90 (3.51), Tyr113 (3.92), Lys121 (3.26), Ile122 (3.39), and Phe130 (3.96). This result suggests that the predicted H bond in Fig 7c with Ile122 is a hydrophobic interaction.

Although there are hardly any direct studies on the role of curcumin in human stress response and relating to mental illnesses, Wei et al. 2010 found that curcumin treatment effectively reduced the subacute stress induced by 2 hours of road transport in pigs. This decrease in stress levels was evidenced by reduced serum cortisol concentration, decreased hippocampal nitric oxide (NO) production, and increased brain-derived neurotrophic factor (BDNF) mRNA expression [113]. These results suggest that curcumin could mitigate stress responses in peripheral and central systems, indicating its potential application in managing stress in animal and human contexts. Studies have also shown that curcumin has antidepressant-like effects in animal models [114–116], positioning it as a potential therapeutic agent for managing depressive symptoms associated with stress. Its ability to modulate neuroprotective mechanisms and neurochemical pathways, as demonstrated by its anti-depressant-like effects, suggests it may have utility in mitigating the negative impact of stress on mental health. By targeting key pathways and proteins involved in stress response and mood regulation, curcumin could offer a novel approach to the prevention and treatment of stress-related mental illnesses such as depression.

Pi (π), stacking interaction was also observed with SAFit2 interaction with Tyr113 and Phe774 (S1 Fig). Hydrophobic interactions were formed with Ile87, Phe130, Phe67, and Val86. S1b Fig categorized Tyr113 as a hydrogen bond with a bond length of 2.57. It also showed H bond formation with Gly84 and Phe774. Pumicalagin also showed pi (π) stacking interaction with Phe77 (S2 Fig). Hydrogen bond formation with corresponding bond length in brackets was observed with Tyr113 (2.77), Ser118 (2.82), Gln85 (3.13, 2.15, 3.17), Val78 (2.90), Pro76 (2.92, 3.01), Gln75 (2.72, 2.71), and Ser70 (2.50, 2.66).

In S3 Fig, rapamycin formed hydrogen bonds with Ile87 and Asp68 (S3b Fig, Ser69 (bond length of 3.15) and Tyr57 (bond length of 2.61) (S3d Fig). Hydrophobic interactions were formed with Tyr113, Lys121, and Phe77. Tacrolimus (S4 Fig) and ascomycin (S5 Fig) had similar observations. This is unsurprising as tacrolimus and pimecrolimus are derivatives of ascomycin [117]. Both had hydrogen bond formation with Asp68 and Gln85 with bond lengths of 3.32 and 2.51 for tacrolimus and 3.17 and 2.53 for ascomycin, respectively (S4d Fig). Additional H bond formation with Ser118, Try113, and Val 86 is also observed from the Molegro images. Hydrophobic interactions were formed with Trp90 and Lys121 for both, with an addition of Tyr57 for tacrolimus. Pimecrolimus formed hydrogen bonds with Val78 with a bond distance of 3.21 and hydrophobic interaction with Phe77 (S6 Fig).

In our results, tyrosine (Tyr113) consistently forms hydrogen bonds or hydrophobic interactions with each of the mentioned ligands, indicating its significance in the ligand-protein interactions across these molecular complexes. In the study by Gopalakrishnan et al. 2012, Tyr113 emerges as a critical player, forming a hydrogen bond with the ligand’s C8-carbonyl and influencing ligand interactions [9]. This is important as tyrosine is involved in synthesizing neurotransmitters, such as dopamine, norepinephrine, and epinephrine, that are critical for proper communication between nerve cells in the brain [118]. Under stress, tyrosine contributes to the production of stress neurotransmitters like epinephrine and norepinephrine [118]. Dopamine is associated with mood regulation, reward, and motivation [119]. Mental illnesses, such as bipolar disorder and other psychiatric conditions, have been linked to excessive or dysregulated dopamine [120]. This emphasizes the significance of tyrosine in stress response.

In Gopalakrishnan et al. 2012 study, the 80s loop (Ser118-Ile122) forms a significant pocket (occupied by the ligand’s tert-pentyl group) crucial for ligand binding. Hydrogen bonding patterns involving Ile87 and Tyr113 highlight precise interactions. Their comparisons with the FK506 complex reveal structural insights, emphasizing the importance of Tyr113 and the 80s loop in consistent ligand recognition and binding to FKBP5. Their study also implicated key amino acid residues: Phe77, Asp68, and Trp90 [121]. These amino acid residues were also involved in our study’s interactions, highlighting their importance during the interaction. The ligand interaction with the shortest hydrogen bond length in our study is the interaction between SAFit2 and Tyr113 with a bond length of 2.57 Å, followed by pumicalagin and Tyr113 with a bond length of 2.77 Å, ruscogenin-Asp68 at a bond length of 2.80 Å, neoruscogenin-Ser118 (bond length of 2.98 Å), curcumin- Ser118 (bond length of 2.78 Å), and apigenin-Gln85 (bond length of 2.94 Å) in S7 Fig.

### Molecular dynamic simulation analysis

Molecular dynamic simulation of the best-interacting ligands for BBB (ruscogenin, neoruscogenin) and gastrointestinal activity (curcumin) confirms effective interactions with high negative binding energy. The binding free energy (ΔG) of ruscogenin associated with FKBP5 protein over the simulation time is -30.41 kcal/mol, -31.78 kcal/mol for neoruscogenin, and -27.6 kcal/mol for curcumin (Table 6). This is calculated using the Molecular Mechanics Poisson-Boltzmann Surface Area (MMPBSA). The total binding free energy (ΔG) is calculated using G =  G_gas_ +  G_solv_. In a similar study by Bager et al. 2021, the MMPBSA binding free energy between SAFit2 and FKBP5 is -21.58 kcal/mol [122]. Compared with our study findings, ruscogenin, neoruscogenin, and curcumin performed better than SAFit2 in their study. However, it is important to note the potential differences in computational methodology.

**Table 6:** MMPBSA delta energies in kcal/mol.

Ligand	VDWAALS^a^	EEL^b^	EPB^c^	ENPOLAR^d^	GGAS^e^	GSOLV^f^	Total (kcal/mol)
Ruscogenin	-41.05	-11.71	25.67	-3.32	-52.76	22.35	-30.41
Neoruscogenin	-44.62	-5.98	22.68	-3.86	-50.60	18.82	-31.78
Curcumin	-36.92	-4.91	17.64	-3.41	-41.82	14.22	-27.6

^a^van der Waals Binding Energy.

^b^Electrostatic Energy.

^c^Polar Solvation Energy.

^d^Nonpolar Solvation Energy.

^e^Gas Phase Energy.

^f^Solvation Free Energy.

**Fig 8:**
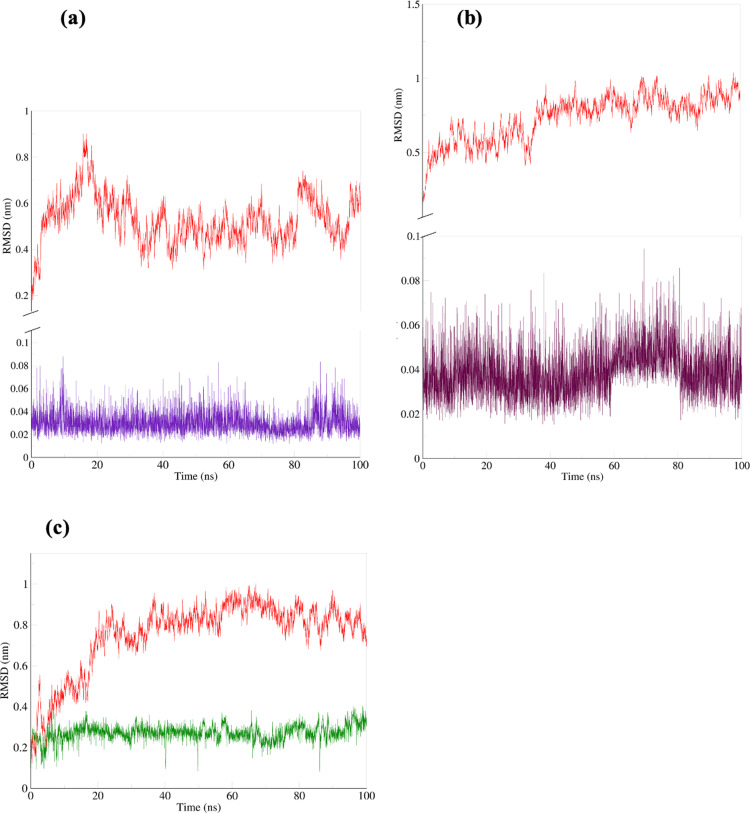
Root-mean-square-deviation (RSMD) in nanometers (nm) of protein and ligand plotted against time in nanoseconds (ns). (a) ruscogenin-FKBP5 system (b) neoruscogenin-FKBP5 system (c) curcumin-FKBP5 system. The protein backbone is represented in red, ruscogenin in violet, neoruscogenin in maroon, and curcumin in green.

**Fig 9:**
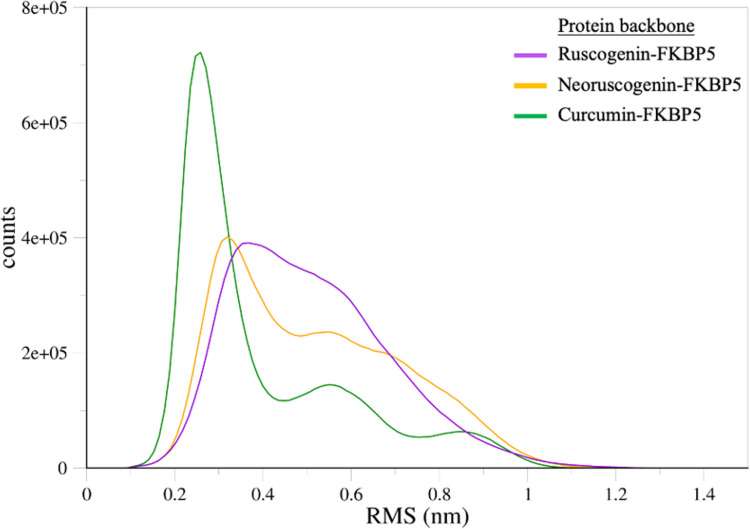
RSM Distribution of protein fluctuation. The x-axis represents the root mean square deviations in nanometers (nm), and the y-axis in counts represents the frequency or occurrence of specific RMS deviation values throughout the simulation.

The root-mean-square-deviation (RMSD) shows how much a system deviates from its original conformation during simulation, indicating its stability [123]. Low RMSD and root-mean-square-fluctuation (RMSF) values indicate interaction stability, and high values indicate structural fluctuation [124,125]. RMSD values less than 2.0 A are considered best, and values between 2.0 A and 3.0 A are deemed acceptable [126]. In Fig 8, the RMSD of our protein backbone is roughly stable in all simulations from 25 ns with only slight variation in distances (nm). The RMSD increased initially between 0 ns and 25 ns before stabilizing. The RMSD of ruscogenin in Fig 9a is stabilized between 0.02 nm and 0.04 nm throughout the simulation. Neoruscogenin (Fig 9b) is stabilized between 0.02 and 0.05 nm with a slight deviation between 60 and 80 nm before returning to the stabilized position. For curcumin (Fig 9c), stabilization is observed between 0.2 and 0.35 nm throughout the simulation.

For a stable simulation, the ligand should stay in the binding pocket, indicated by a very low ligand RMSD. Our results show a lower RMSD value than the protein backbone, indicating a stable binding. The protein backbone’s high RMSD values in the 0.8 to 1.0 nm range likely reflect the protein’s conformational flexibility. Large proteins such as FKBP5 [83] exhibit inherent flexibility due to their size and complex structure, particularly in regions like loops, termini, or domain interfaces [127]. This flexibility leads to significant deviations in the protein backbone, as measured by RMSD (Root Mean Square Deviation). These deviations are natural and reflect the protein’s dynamism, especially when adapting to ligand binding. Ligand-induced structural rearrangements, including small local changes, can contribute to high RMSD values when summed across many residues [128,129]. Similar to our study, in the study by Barge et al. 2021, on *in silico* investigation of potential inhibitors for FKBP5, their 14 hit compounds in complex with FKBP5 had a protein backbone RSMD up to 1.5nm [122]. Their top 5 hit compounds had a protein RSMD between 0.1 and 1.16nm. In our simulation, the protein reached equilibrium at approximately 30 ns, after which the RMSD plateaued, indicating a stable conformation. This stability suggests that the observed high RMSD values are not due to instability but are instead reflective of the protein’s flexibility and structural adjustments to ligand binding. To further substantiate the stability and integrity of the protein-ligand complex, additional analyses (Root Mean Square Fluctuation, Solvent Accessible Surface Area, and Radius of Gyration) were performed. These metrics confirm that the protein-ligand complexes remain stable despite the high RMSD values, which primarily reflect natural flexibility and conformational adjustments. Therefore, the high RMSD values should not be interpreted as instability but as an expected feature of a flexible protein undergoing ligand-induced dynamics.

Fig 9 shows the RMS (Root Mean Square) Distribution of atomic fluctuations or deviations in nanometers (nm) that shows the flexibility of the different parts of the protein backbone. Regions of the protein with higher RMSD values fluctuate more, indicating greater flexibility [130]. The Curcumin-FKBP5 complex displays a sharp and distinct peak around 0.2 nm to 0.25 nm, with a narrower distribution than the other two complexes. This indicates that curcumin binding restricts the conformational flexibility of the protein backbone, stabilizing specific structural states. The reduced heterogeneity in the RMS distribution suggests that curcumin induces a more rigid and defined conformation in FKBP5. The neoruscogenin-FKBP5 complex exhibits a relatively broad distribution with a peak centered around 0.3 nm. This indicates that the protein retains significant conformational flexibility upon binding neoruscogenin. The broad nature of the distribution suggests effective but less rigid binding interactions. The ruscogenin-FKBP5 complex shows a broader and less pronounced peak compared to neoruscogenin-FKBP5. This broader distribution suggests that ruscogenin binding increases the conformational sampling of the protein backbone, leading to enhanced flexibility. Such increased dynamics may reflect diverse interactions or binding modes between ruscogenin and FKBP5. The differential effects of ligand binding on FKBP5 flexibility highlight the unique roles of loops, turns, and coils (indicated by regions with flexibility) in modulating protein dynamics. Ruscogenin and neoruscogenin maintain or enhance the flexibility of these regions, while curcumin imposes structural constraints, potentially stabilizing functionally relevant conformations. The observance of flexible regions corresponding to loops, turns, or coils in a protein structure indicates that amino acids are present in active binding sites or near active binding site regions [131,132]. Barge et al. 2021, highlighted that ligand binding induces flexibility and significant conformational changes in FKBP5, with dynamic shifts in secondary structures near active site regions and terminal areas [122]. Therefore, the high RMSD values in Fig 8 reflect the flexibility of dynamic regions that facilitate the ligand interactions.

**Fig 10:**
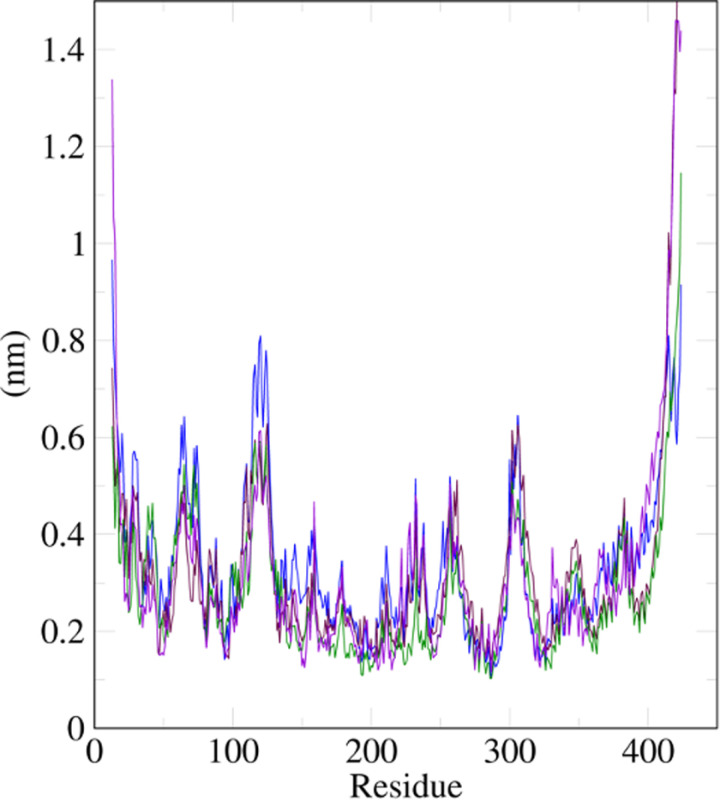
Root-mean-square-fluctuation (RSMF) in nanometers (nm) of protein plotted against the amino acid residues. Ruscogenin is represented in violet, neoruscogenin in maroon, and curcumin in green.

The root-mean-square-fluctuation (RMSF) is used to assess the fluctuation of the protein backbone with respect to the ligand, with a high value indicating fluctuation. The RMSF of our system for all complexes had no significant fluctuations and remained stable between 0.25 nm and 0.7 nm with varying peak regions at different amino acid residues (Fig 10). This is expected as all systems had the same FKBP5 protein.

**Fig 11:**
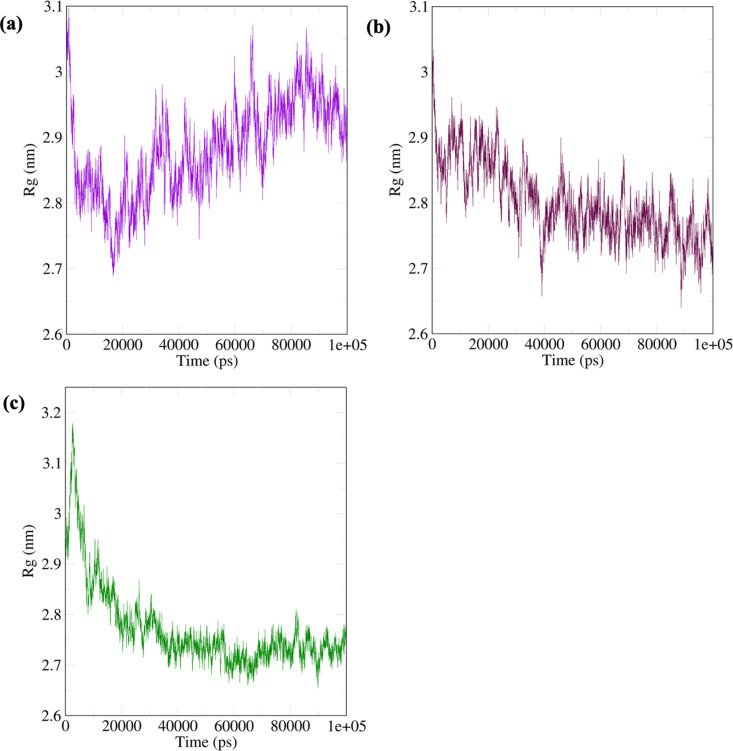
The radius of Gyration (Rg) of the systems. (a) ruscogenin-FKBP5 system (b) neoruscogenin-FKBP5 system (c) curcumin-FKBP5 system.

The radius of gyration (Rg) gives an overview of the conformational state of the complex [133]. In Fig 11a, the Rg of the ruscogenin-FKBP5 system increased with time between 2.7 nm and 3.1 nm. The increasing Rg suggests a more extended or flexible conformation, indicating a conformational change leading to a larger overall complex size. For the neoruscogenin-FKBP5 and curcumin-FKBP5 systems (Fig 11b and 11c), Rg decreased, suggesting a more compact or constricted conformation, indicating a structural change that reduces the overall size of the complex.

**Fig 12:**
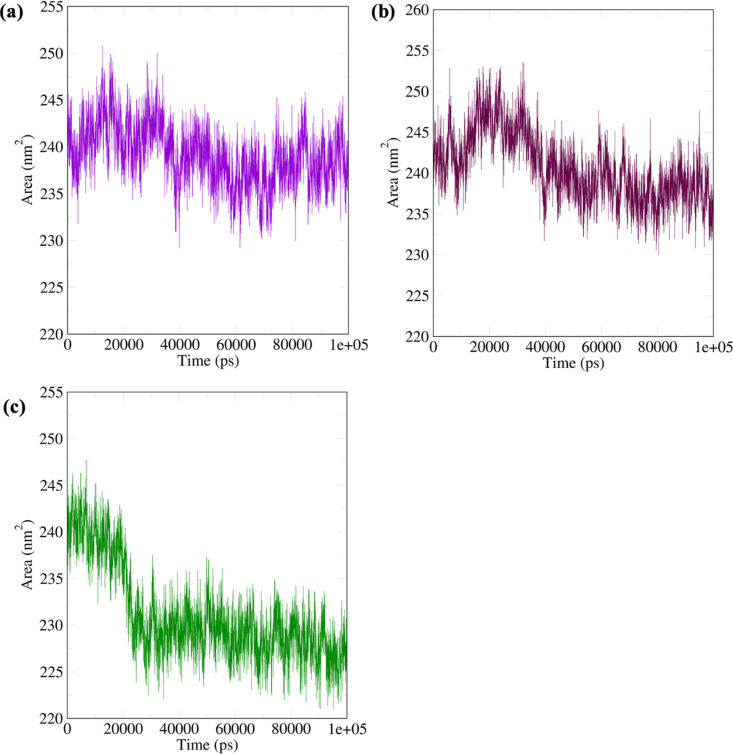
Solvent accessible surface area (SASA) of the systems. (a) ruscogenin-FKBP5 system (b) neoruscogenin-FKBP5 system (c) curcumin-FKBP5 system.

Solvent accessible surface area (SASA) represents the surface areas of the protein accessible to a solvent and gives information about the system’s conformational changes. Fig 12a for the ruscogenin-FKBP5 complex shows stability between 235 and 245 nm^2^; Neoruscogenin-FKBP5 (Fig 12b) is stabilized between 235 and 240 nm^2^. For the curcumin-FKBP5 complex (Fig 12c), stability was achieved between 224 and 235 nm^2^. This equilibrium state suggests that binding the ligands to the protein induces structural changes that result in exposed and buried regions that balance the accessible surface.

**Fig 13:**
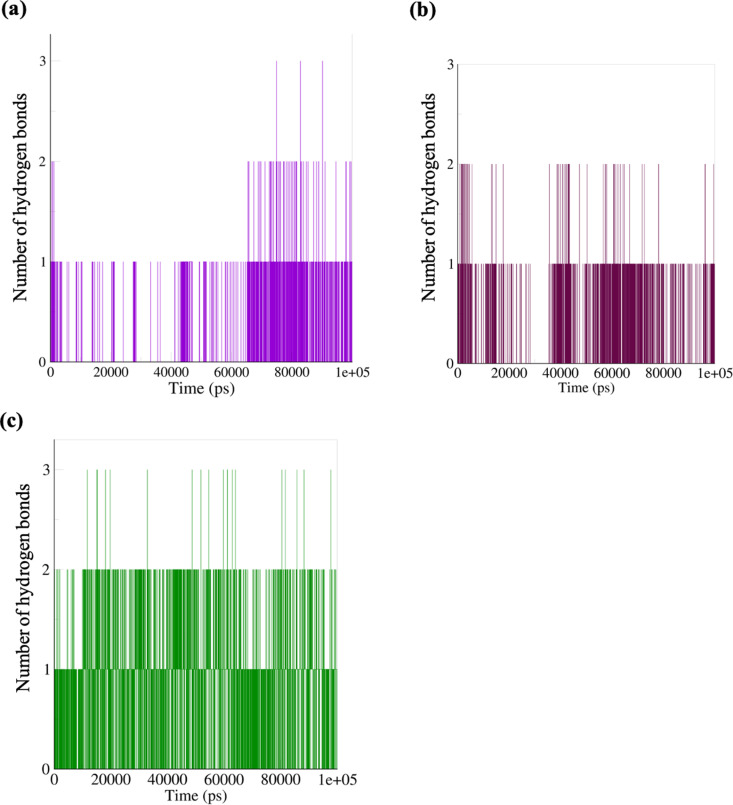
Ligand hydrogen bond formation. (a) ruscogenin (b) neoruscogenin (c) curcumin.

The average hydrogen bond found for the simulation is 2 for the ruscogenin-FKBP5 complex (Fig 13a), mainly towards the end of the simulation after 6000 ps. The neoruscogenin-FKBP5 complex (Fig 13b) also had 2 hydrogen bonds throughout the simulation. The average hydrogen bond for curcumin-FKBP5 (Fig 13c) is 3, but mainly 2 hydrogen bonds sustaining the complex throughout the simulation. These indicate that the ligands require, on average, 2 hydrogen bonds to be stably held in the protein’s active pocket throughout the simulation. In calculating the hydrogen bonds between the ligands and the protein, 587 donors and 1182 acceptors were observed.

## Conclusions and future perspectives

To the best of our knowledge, this is the first study that presents the efficient interactions between ascomycin, pimecrolimus, curcumin, apigenin, ruscogenin, neoruscogenin, and pumicalagin with the human FKBP5 protein. Notably, ruscogenin and neoruscogenin can permeate the Blood-Brain Barrier (BBB) and possess favorable gastrointestinal absorption, while curcumin demonstrates suitable absorption properties. All three ligands exhibit excellent drug properties, adhering to Lipinski’s rule, and efficient interactions in ligand-FKBP5 complexes concerning binding energy and conformational changes. Ruscogenin and neoruscogenin had favorable safety profiles and low toxicity, emerging as promising therapeutic candidates for FKBP5 targeting. Toxicity insights for all ligands provide critical guidance for optimizing design, dosage, and delivery to enhance safety and efficacy. Ruscogenin, neoruscogenin, and curcumin interacted better with the FKBP5 protein than the reference ligands (SAFit2, rapamycin, tacrolimus). Molecular dynamic simulation analysis (RMSD, RMSF, Rg, SASA, ligand-protein H bond, MMPBSA binding energy) validated these ligand-protein interactions. Consequently, our findings propose ruscogenin, neoruscogenin, and curcumin as potential therapeutic molecules for the modulation of FKBP5-related mental illnesses. However, further functional studies, including in vivo experiments, X-ray crystallography, Enzyme-Linked Immunosorbent Assay (ELISA), cell-based assays, and nuclear magnetic resonance (NMR), are necessary to comprehensively assess their therapeutic, cytotoxic, inhibitory, and interaction abilities.

In stress-related bipolar disorder, the identified ligands, ruscogenin, neoruscogenin, and curcumin, hold promise as candidates for modulating *FKBP5* gene expression or protein levels and regulating stress response; thus, they may have therapeutic implications for bipolar disorder and other mental illnesses.

## Supporting information

S1 Fig SAFit2-FKBP5 complex visualization. (a) 3D structural orientation of the complex from Protein Plus (b) 2D binding pose interaction of the complex from Protein Plus. Black dash lines represent H bonds, green lines represent hydrophobic interaction, green dot represents π-π stacking (c) 3D arrangement of the complex from Molegro Molecular Viewer. Blue dash lines represent H bonds (d) 2D visualization of the interaction from LigPlot. The green text represents the interacting residue, and the green dashed lines represent the H bond.
(DOCX)

S2 Fig Pumicalagin-FKBP5 complex visualization. (a) 3D structural orientation of the complex from Protein Plus (b) 2D binding pose interaction of the complex from Protein Plus. Black dash lines represent H bonds, green lines represent hydrophobic interaction, green dot represents π-π stacking (c) 2D visualization of the interaction from LigPlot. The green text represents the interacting residue, and the green dashed lines represent the H bond.
(DOCX)

S3 Fig Rapamycin-FKBP5 complex visualization. (a) 3D structural orientation of the complex from Protein Plus (b) 2D binding pose interaction of the complex from Protein Plus. Black dash lines represent H bonds, green lines represent hydrophobic interaction (c) 3D arrangement of the complex from Molegro Molecular Viewer. Blue dash lines represent H bonds (d) 2D visualization of the interaction from LigPlot. The green text represents the interacting residue, and the green dashed lines represent the H bond.
(DOCX)

S4 Fig Tacrolimus-FKBP5 complex visualization. (a) 3D structural orientation of the complex from Protein Plus (b) 2D binding pose interaction of the complex from Protein Plus. Black dash lines represent H bonds, green lines represent hydrophobic interaction (c) 3D arrangement of the complex from Molegro Molecular Viewer. Blue dash lines represent H bonds (d) 2D visualization of the interaction from LigPlot. The green text represents the interacting residue, and the green dashed lines represent the H bond.
(DOCX)

S5 Fig Ascomycin-FKBP5 complex visualization. (a) 3D structural orientation of the complex from Protein Plus (b) 2D binding pose interaction of the complex from Protein Plus. Black dash lines represent H bonds, green lines represent hydrophobic interaction (c) 3D arrangement of the complex from Molegro Molecular Viewer. Blue dash lines represent H bonds (d) 2D visualization of the interaction from LigPlot. The green text represents the interacting residue, and the green dashed lines represent the H bond.
(DOCX)

S6 Fig Pimecrolimus-FKBP5 complex visualization. (a) 3D structural orientation of the complex from Protein Plus (b) 2D binding pose interaction of the complex from Protein Plus. Black dash lines represent H bonds, green lines represent hydrophobic interaction (c) 3D arrangement of the complex from Molegro Molecular Viewer. Blue dash lines represent H bonds (d) 2D visualization of the interaction from LigPlot. The green text represents the interacting residue, and the green dashed lines represent the H bond.
(DOCX)

S7 Fig Apigenin-FKBP5 complex visualization. (a) 3D structural orientation of the complex from Protein Plus (b) 2D binding pose interaction of the complex from Protein Plus. Black dash lines represent H bonds, and the green line represents hydrophobic interaction (c) 3D arrangement of the complex from Molegro Molecular Viewer. Blue dash lines represent H bonds (d) 2D visualization of the interaction from LigPlot. The green text represents the interacting residue, and the green dashed lines represent the H bond.
(DOCX)

S1 Table SwissADME of 10 best ligands. (XLSX)
