# Supplementary material for: In silico identification of novel ligands targeting stress-related human FKBP5 protein in mental disorders
Source: PLoS One. 2025 Mar 17;20(3):e0320017. doi: 10.1371/journal.pone.0320017 (PMC11913304; doi:10.1371/journal.pone.0320017)
Supplement: S3 Fig — (a) 3D structural orientation of the complex from Protein Plus (b) 2D binding pose interaction of the complex from Protein Plus. Black dash lines represent H bonds, green lines represent hydrophobic interaction (c) 3D arrangement of the complex from Molegro Molecular Viewer. Blue dash lines represent H bonds (d) 2D visualization of the interaction from LigPlot. The green text represents the interacting residue, and the green dashed lines represent the H bond. (DOCX) [file pone.0320017.s003.docx]

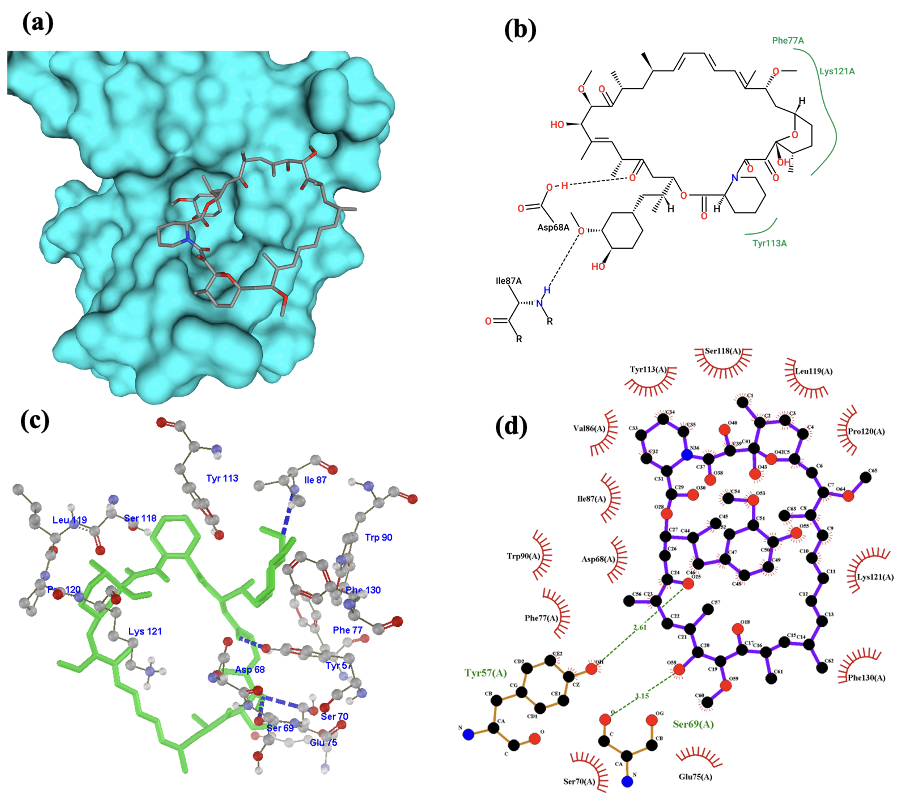


**Figure S3. Rapamycin-FKBP5 complex visualization.** **(a)** 3D structural orientation of the complex from Protein Plus **(b)** 2D binding pose interaction of the complex from Protein Plus. Black dash lines represent H bonds, green lines represent hydrophobic interaction **(c)** 3D arrangement of the complex from Molegro Molecular Viewer. Blue dash lines represent H bonds **(d)** 2D visualization of the interaction from LigPlot. The green text represents the interacting residue, and the green dashed lines represent the H bond.
